# Choice of speciality amongst first-year medical students in the Nelson R. Mandela School of Medicine, University of KwaZulu-Natal

**DOI:** 10.4102/phcfm.v5i1.513

**Published:** 2013-06-28

**Authors:** Onyemaechi O. Azu, Edwin Naidu, Jesse Naidu

**Affiliations:** 1 Discipline of Clinical Anatomy, Nelson R. Mandela School of Medicine, University of KwaZulu-Natal, South Africa

## Abstract

**Background:**

Trends in career choice amongst medical graduates have considerable implications for the percentage of the workforce available for training.

**Objective:**

To investigate and review factors affecting career choice by undergraduate first-year medical students.

**Method:**

This was a cross-sectional study using a closed-ended, semi-structured survey instrument. Two hundred and four questionnaires were administered to all first-year medical students at the Nelson R. Mandela School of Medicine in the first term of the 2012 academic session.

**Results:**

The questionnaire was completed by 167 out of 204 students (81.8% response rate). Most of the respondents were South Africans (91%) and blacks (72%), with a higher proportion of women to men (2:1). The majority (86%) intended to undertake their postgraduate training in surgical specialties (53%), general surgery (50%) and cardiology (46%). Few were interested in an academic career in basic sciences (27.6%), either because they were not interested in research and/or teaching (48%), not clinically-orientated (20%), or found it to be an unattractive choice (12.3%). The top perceived career-related factors favouring choice of speciality were personal interest and benefits to patients as many (83%) respondents still viewed the medical profession as having a bright future in South Africa.

**Conclusions:**

Our study highlighted the fact that self and patient interests were strong determinants of speciality choices by the students and the role of parents and practice in rural areas were considered least as potential influencing factors. This would appear to be a good indicator that the healthcare sector may be boosted in the future by doctors who are wholeheartedly committed to the service of the communities with the greatest disease burden.

## Abstrait

**Le choix des spécialités chez les étudiants en première année de médecine à l’école de médecine Nelson R. Mandela, de l'université du Kwazulu Natal**

**Contexte:**

Les tendances en matière de choix de carrière chez les étudiants en médecine ont des répercussions très importantes sur le pourcentage du personnel disponible pour les formations.

**Objectif:**

Enquêter et examiner les facteurs affectant le choix de carrière des étudiants en première année de médecine.

**Méthode:**

Il s'agissait d'une étude transversale utilisant un instrument d’étude à questions ouvertes, semi-structuré. Deux cent dix questionnaires ont été remis à tous les étudiants en première année de médecine de l’école de médecine Nelson R. Mandela au premier semestre de l'année universitaire 2012.

**Résultats:**

Le questionnaire a été complété par 167 des 204 étudiants (taux de réponse de (81.8%). La plupart des sondés étaient Sud-africains (91%) et Noirs (72%), la proportion des femmes par rapport aux hommes étant plus élevée (2/1). La majorité (86%) souhaitait suivre la formation de troisième cycle dans des spécialités chirurgicales (53%), en chirurgie générale (50%) et en cardiologie (46%). Peu d'entre eux étaient intéressés par une carrière universitaire en sciences fondamentales (27.6%), parce qu'ils n’étaient pas intéressés par la recherche et/ou l'enseignement (48%), qu.ils n.étaient pas orientés vers l'aspect clinique (20%), ou qu'ils considéraient cela comme un choix peu séduisant (12.3%). Les premiers facteurs liées à la carrière perçus comme influençant le choix de la spécialité étaient l.intérêt personnel et les bénéfices pour les patients, bon nombre de sondés (83%) considérant toujours que la profession médicale avait de beaux jours devant elle en Afrique du Sud.

**Conclusions:**

Notre étude a souligné le fait que les intérêts personnels et ceux des patients constituaient de solides déterminants des choix de spécialité des étudiants et que le rôle des parents et de la pratique en milieu rural étaient moins considérés comme des facteurs d'influence potentiels. Ceci semble être un bon indicateur du fait que le secteur des soins de santé pourrait être stimulé à l'avenir par des médecins qui sont engagés de tout cœur au service des communautés les plus affectées par la maladie.

## Introduction

Medical education has tried to keep up with the changing health system over the years, despite its regional variability.^1, 2^ Career choices of medical students have therefore become a topic that attracts the interest of medical educators as well as health service providers globally.^3^ Because these choices affect significantly the availability and distribution of medical personnel as well as the quality of service rendered by the health system,^4, 5^ it can become problematic when certain specialities may have the difficulty of meeting up with the requisite number of experts at critical levels of healthcare training and delivery.^6^ Studies carried out by researchers have indicated that predictors of these changing trends in choice of speciality include demographic variables,^7^ lifestyle variables,^1, 2, 8^ financial issues^1, 9^ and institutional peculiarities,^10^ amongst others.

Although researchers have approached the topic in a number of ways, most approaches have tended to focus on narrow elements of the choice, such as the effect of programmes or curricula.^11^ From a global perspective, however, there have been significant changes in medical students’ career choices in countries such as USA,^5^ South Africa^12^ and Australia.^13^ Consequently, medical students who are deciding which specialities to enter do not always choose the one they actually prefer.^14^ Again, the emphasis in medicine has shifted toward increasing the number of physicians who choose certain specialities over others, in order to balance region- and/or country-specific needs. This trend creates an unfavourable shift that may disadvantage certain specialities of the medical sciences disciplines, leading to a persistent dearth of the experts who are needed to continue the training of medical students in the various tertiary centres across the country.

We undertook this study in order to explore the dynamics of career and medical specialisation decisions and inclinations of first-year medical students at the Nelson R. Mandela School of Medicine, University of KwaZulu-Natal (UKZN). This was done with a view to establishing first-line data on these students and then seeing how and when these career choices are changed (if at all) during their six-year training programme, as well as what factors are associated significantly with these variations. Expected output recommendations would address how they match the need of medical services in the KwaZulu-Natal region and the country as a whole and to discover those factors which may be useful in this connection. In our opinion, there is a paucity of data regarding the continued monitoring of career choices of medical students during their training in both the region and the country as a whole and it is expected that these results may form a significant landmark for policymakers and administrators in the healthcare sector.

## Research method and design

### Setting

This study of a cohort was conducted within the first semester of the year (January to April 2012) at the Nelson R. Mandela School of Medicine, UKZN, Durban. Only first-year medical students were sampled in the survey. Further prospective studies should be done on this cohort through the succeeding five years of their medical studies, taking into cognisance the same period of sampling. A self-administered questionnaire in the English language, which is the official language of the medical training at UKZN, was utilised.

### Ethics approval

Ethics approval for the study was obtained from the Biomedical Research Ethics Committee of UKZN (BE070/12). All respondents were given an information sheet containing details with regard to informed consent for the study and their declaration, as well as whether they would like to receive a summary of the findings of the survey. Confidentiality of the data and respondents was strictly highlighted, as was the fact that there would be the need for follow-ups during their remaining years of training.

### Questionnaire

The content and design of the survey questionnaire was based on guidelines from questionnaires developed by other international researchers^15, 16, 17^ for similar purposes and was modified to suit the objectives of the study. It included structured questions about choices of future long-term career: students were asked to state their choices of future intended career speciality and to state whether their choice was ‘very likely’, ‘unlikely’ or ‘no opinion’. The survey required about 15–20 minutes for completion and consisted of 29 items that explored the following areas:

1. Demographic background characteristics on age, sex, self-reported socioeconomic status, postgraduate study plans.
2. Factors the students considered important in their choice of a speciality (rating choice of ‘very important’, ‘important’ or ‘not important’).
3. The degree to which the students had considered possible careers amongst various specialities.
4. The extent to which students found various specialities attractive enough to build a career on.

### Analysis

Data generated were recorded into SPSS (Statistical Package for the Social Sciences) version 19.0 of 2010 (IBM SPSS Inc., Chicago, IL, USA), then analysed and presented using the appropriate statistical format. Summary statistics have been presented as simple frequencies and percentages. Levels of association were tested using the Chi-square test, with a *p*-value of <0.05 being considered significant statistically.

## Results

Questionnaires were completed and returned by 167 of the 204 students in the first year MBChB programme – a response rate of 81.8%. Sixty-nine per cent were women and 31% men; 75% were between 18 and 19 years of age and the majority (147; 91%) were South African citizens. The majority of the respondents (72.7%) were blacks, never married (98%) and Christian (81%) (Table 1).

**TABLE 1:** Demographics of the 2012 first-year class (*n* = 163).

Variables	Characteristics	*n*	%
**Gender**	Male	49	30.6
	Female	111	69.4
**Age**	≤ 17	8	5.0
	18	76	47.2
	19	44	27.3
	20	8	5.0
	≥ 21	25	15.5
**Race as used in South African Census**	Black	117	72.7
	Coloured	14	8.7
	Indian	27	16.8
	White	3	1.9
**Citizenship**	South African	147	90.7
	Other African country	11	6.8
	Europe	0	0.0
	America	0	0.0
	Other	4	2.5
**First language at home**	English	41	27.5
	Afrikaans	2	1.3
	African	61	40.9
	Indian	1	0.7
	Other	44	29.5
**Marital status**	Single (never married)	159	98.1
	Married/Engaged	2	1.2
	Divorced	1	0.6
**Religion**	Christian	130	81.3
	Moslem	11	6.9
	Hindu	14	8.8
	Other/Atheist/Agnostic	5	3.1

More than half of the respondents (53%) were from middle-income homes and the majority (88%) had no family responsibilities. Interestingly, a high proportion of respondents had no siblings (59%) or family in the medical profession (61%) (Table 2).

**TABLE 2:** Home and family influences of respondents****.

Variables	Characteristics	*n*	%
**Socioeconomic status**	Low	67	42.4
	Middle	84	53.2
	High	7	4.4
**Family situation**	Brothers or sisters older	49	32.5
	Brother or sister younger	48	31.8
	Sibling older and younger	57	37.7
**Family responsibilities**	None	141	88.2
	Sole breadwinner	5	3.1
	Caring for parents	9	5.6
	Caring for sibling/s	5	3.1
**Siblings/Family who are**	In the medical profession	6	5.2
	Academics in tertiary institutions	42	36.2
	Business other than medicine	68	58.6
**Friends and/or other family who are**	In the medical profession	66	60.0
	Academics in tertiary institutions	40	36.4
	Business other than medicine	67	60.9

Only 16% of respondents had previous tertiary education prior to entering medical school and personal interest (94%) ranked the highest as their reason for undertaking a medical career. A mere 27% had received substantial career guidance from their teachers compared with a majority of over 54% who had had little or no guidance (Table 3).

**TABLE 3:** Educational profile and career guidance of respondents.

Variables	Characteristics	*n*	%
**Subjects taken in high school**	Mathematics	139	85.3
	Physics	137	84.0
	Chemistry	134	82.2
	Biology/Life Sciences	139	85.3
	English/Afrikaans	137	84.0
**Previous tertiary academic studies**	Yes	25	15.9
	No	132	84.1
**Reason for entering medical school**	Personal interest	149	94.3
	Mentorship by teacher/guardian	3	1.9
	Parental influence	12	7.6
	You were ‘forced’ to do medicine	0	0.0
**Career guidance from school**	Little guidance	65	40.4
	No guidance	22	13.7
	Informal	31	19.3
	Substantial	43	26.7

### Intentions of respondents after graduation

Most respondents (*n* = 139; 85.8%) intended to specialise, 2 (1.2%) did not and 21 (13.0%) were not sure. Of those who intended to specialise, 81 (52%) did not intend to pursue an academic career in the basic sciences because they were either not interested in teaching and/or research (48%), were not clinically orientated (20%), or found it to be an unattractive choice (12.3%). Only about 28.5% of the students intended to pursue their postgraduate studies within South Africa whereas 22% indicated ‘abroad’ as being their preferred choice. About 48% were still undecided as to their intended geographical postgraduate destination. Students’ satisfaction of the medical profession in South Africa was measured at 48%, with 57.7% choosing ‘Sense of self-achievement’ as being a reason for the bright future of the profession (Table 4).

**TABLE 4:** Intentions of respondents upon graduating.

Variables	Characteristics	*n*	%
**Satisfaction with medical profession in South Africa**	Satisfied	68	42.0
	Not satisfied	48	29.6
	Undecided	45	27.8
**Postgraduate study plans**	Inland	45	28.5
	Abroad	35	22.2
	Undecided	76	48.1
**Preferred sector of work**	Urban	65	44.4
	Rural	37	25.5
	Abroad	21	15.0
	No preference	30	19.6
**Desire to specialise**	Yes	139	85.8
	No	2	1.2
	Not sure	21	13.0
**Intention to specialise in any basic sciences**	Yes	43	27.6
	No	81	51.9
	Not sure	32	20.5
**Reason not to specialise in any basic sciences**	Unattractive	8	12.3
	Poor remuneration	2	3.1
	Not clinically orientated	13	20.0
	Not interested in teaching/research	31	47.7
	Not sure	16	24.6
**Students’ view of their future after medical school**	Bright	131	82.9
	Uncertain	15	9.5
	Negative	1	0.6
	No idea	11	7.0
**Reasons for 'bright future’**	Job-related factors	55	42.3
	Personal attitude	60	46.2
	Sense of achievement	75	57.7

### Speciality choice

The three most-preferred specialities were surgical specialities (53%), general surgery (50%) and cardiology (46%). Again, only 21% chose the basic sciences as an intended area in which to specialise, whilst a mere 17% intended to specialise in psychiatry (Table 5).

**TABLE 5:** Future preferences for intended speciality by respondents.

Speciality	No Opinion	Unlikely	Very Likely
Surgical specialist	19.9	27.6	52.6
General Surgery	21.7	28.0	50.3
Cardiology	25.0	28.8	46.2
Paediatrics	25.0	31.4	43.6
General Medicine	26.8	33.8	39.5
Family Medicine	24.4	37.2	38.5
Obstetrics & Gynaecology	29.1	32.9	38.0
Other Non-Surgical	38.1	32.9	29.0
PhD basic science	35.3	44.2	20.5
Psychiatry	28.8	53.8	17.3
Ophthalmology	48.1	46.8	5.1
No preference	65.1	33.0	1.8

### Factors influencing career choice

A list of factors was provided and the students indicated whether they were ‘very important’, ‘important’ or ‘not important’ with regard to their career choice. Overall, the students rated ‘Benefits to patients’ (82.8%) and ‘Personal interest’ (86.5%) as being the most influential factor that contributed to their career choice. Other important influences were ‘Love for speciality’ (79.8%) and ‘Challenge’ (55.5%), whilst ‘Parental influence’ (8.6%) and ‘Prestige’ (12.3%) ranked lowest amongst the factors that influenced their choice of specialities (Table 6).

**TABLE 6:** Reasons for choice of speciality by respondents.

Reasons	Not Important	Important	Very Important
Benefits to patients	1.9	15.3	82.8
Personal Interest	1.9	11.5	86.5
Others (e.g. love)	3.9	16.3	79.7
Challenge	14.2	30.3	55.5
Financial	22.4	48.7	28.8
Lifestyle	23.1	48.1	28.8
Working Lifestyle	45.5	38.5	16.0
Working hours	45.5	38.5	16.0
Like urban area	53.6	25.8	20.5
Status/Prestige	55.5	32.3	12.3
Like Rural area	56.3	24.3	19.4
Parental Influence	74.8	16.5	8.6

## Ethical considerations

Prior to administration of the research instrument, ethical approval was obtained from the Biomedical Research Ethics Committee (BREC) of the University of KwaZulu-Natal (BE070/12). The study posed no direct or indirect harm to the students involved in the study. Participants were given an information sheet of informed consent for the study prior to the questionnaire which included information that this survey was purely voluntary.

## Trustworthiness

This study has been advanced in the evolving field of medical sciences education, thus the output article will be peer-reviewed by external experts and eventually published for reference by others. All literature sources and data as well as materials utilised and represented by the authors in the paper are credible, dependable and can be verified.

## Discussion

Although there have been studies on the career plans of final-year medical students in South Africa,^12^ Pakistan,^2, 18^ Nigeria^19, 20^ and the United States of America,^5^ there is a paucity of information regarding studies of career choices of first-year medical students and factors that precipitate changes in these choices (if at all) during the course of medical training in South Africa. This is particularly critical because sub-Saharan Africa has been identified as the region worst affected by an exodus of trained medical personnel with the attendant shortage of a healthcare workforce.^21^

The proportion of students in our study who intended to specialise after graduation (83%) is lower than has been reported by Burch *et al*.^21^ (90%) and Dambisya^22^ (90%), but close to a similar study in Malawi.^23^ These studies, however, were carried out amongst medical students in their final year and/or across the curriculum, but we have focused specifically on ‘fresher’ medical students. Specialisation preferences amongst our respondents followed the usual trends from other studies in South Africa and Nigeria amongst medical students, in that surgical specialties are most preferred. The ratio of male:female students in our survey was 1:2, with the balance tilting towards a higher female enrolment in the medical school of UKZN. This is similar to trends in the United States, where female applicants to medical schools outnumber the males,^24^ but is contrary to the male-dominated enrolment seen in most Nigerian medical schools.^25^ The reason for this imbalance is yet to be identified but is most likely related to the South African policy of encouraging female enrolment in academic institutions as well as in jobs. If we reflect on the fact that only about 5% of the respondents come from families with parents and/or siblings in the medical profession and more than 94% chose medicine because of personal interest, it becomes intriguing to ascertain what factors might have been responsible for this reasoning. It is, however, another positive indication that the bulk of the students enrolled at UKZN medical school may actually have seen themselves as eventual ‘caregivers and doctors’, desirous of changing the healthcare delivery within the region. It may also translate, albeit contrarily, to the notion that parents often view their children's career accomplishments as a reflection on themselves and as material for the construction of meaning in their own lives.^25^

What we found regarding career guidance and/or mentoring was similar to that reported by Dambisya,^22^ with a consistently high proportion of respondents having little or no career guidance prior to commencement of their medical training. This then implies that the majority of students enter medical school without any prior knowledge of what is expected of them, making room for uncertainties and possibly contributing to skewed career choices.

The trend of UKZN medical students intending to work abroad is low and this encouraging index implies that the majority would be retained locally to pursue their desired postgraduate career and/or assist in the healthcare delivery within the sector. Given the fact that there are sufficient postgraduate training capacities in the various fields of medicine in the university and its associated support institutions, it is not unlikely that this might have contributed to this trend, as was reported previously by Burch *et al*.^21^ We further allude to the fact that there is a generally positive perception of the medical practice, with many respondents seeing a bright future for the medical profession in South Africa.

Career decisionmaking has been conceptualised as occurring continuously throughout the lifespan of an individual and is not necessarily limited to early adulthood. Individuals may not make only one career decision but, when faced with different life events, may revise their career decisions over time, allowing for possible indecision during these transitions.^26^ For those pursuing careers in medicine, there are many transition points at which indecision can occur. To start with, the individual has to decide to choose medicine as a career, but not long after they begin their education at medical school, questions begin to arise about what medical speciality they will be taking. The decision about speciality choice is revisited as the student progresses through the curriculum and is exposed to different areas of medicine.^27^ Our study therefore sets out to establish first-line data on the career intentions of new intakes in the medical school and the associated background information, as well as to assess changes to these choices (if any) and the factors responsible therefore. This study will build critical data that will become a reference point for policymakers and trainers with the aim of identifying possible manpower gaps within the region and of finding ways to boost the availability and/or distribution of medical personnel in the health sector.

Our findings further confirm that the choice of speciality is a complex decision, strongly influenced by diverse factors (intrinsic and extrinsic) that eventually modulate the final outcome. Although about 39% of the students intend to specialise in family medicine, previous reports from surveys across other African institutions indicate that this is not the case.^12, 19, 20^ Whilst this observation is very encouraging in view of the fact that the region has a high disease burden and is in critical shortage of a healthcare workforce,^28^ the passage of time and other factors will determine eventually if this inclination will not suffer from some level of attrition. Nonetheless, this outcome (in our opinion) should become a strong focus for further intervention by way of support and/or counseling by academics involved in the training of the students. By so doing, the fear of attrition of students to other ‘high interest’ specialities may be addressed and sufficient workforce retained to tackle local public health issues, because one way in which medical students gain information about speciality options is through their exposure to clinical environments and to physicians they encounter during their medical training.^29^

Given that early in their training medical students may not always have a clear understanding of what it means to be a physician, the possibility that career aspirations may be guided toward a particular field becomes even more apparent. It is against this background that our results become even more compelling, especially with regard to the aspect of developing human capacities for training in the medical sciences. Mullan *et al*.^30^ highlighted this decline in human resources for teaching in medical schools across sub-Saharan Africa, showing that there is also a consistent loss of teaching staff personnel to the phenomenon of ‘brain drain’. In this study, the fraction of students intending to pursue an academic career in basic sciences is only 20.5%, with a majority of 53.8% unlikely to undertake this career pathway. Although this proportion may be slightly higher than that reported by other studies,^21, 22, 31^ it is below that reported by Anand *et al*.^32^ The impression this gives is that these students enter medical schools with a preconceived notion which is not being changed as a result of their experience whilst in medical school. Therefore, as teachers of basic sciences, we need to do more to encourage the students by stimulating their interest through relevant activities. Strategies to make an academic career more attractive to medical students have to be explored; this may be another avenue where increased funding could be used to build capacity in the KwaZulu-Natal region (and hence South Africa) so that it is better equipped to address its own needs. In the same vein, this proportion of students intending to pursue their specialisation in the basic sciences remains very encouraging if it is measured relative to the clinical science disciplines (an almost 1:5 ratio). However, whether this ratio will continue to remain unchanged in the succeeding years and upon graduation remains to be seen, but this index is a positive base to be worked upon by concerned authorities and experts.

The KwaZulu-Natal region is predominantly a black-dominated population, which accounts for the preponderance of black students in our survey. This is not unusual, as a similar trend has been observed in a survey by Dambisya^22^ for the Eastern Cape region of South Africa. It would be interesting to know what percentage of these black students will eventually stay in South Africa after their graduation, as it is currently the view that black graduates of South African medical schools are more likely to remain within the country than their white counterparts.^16^

In South Africa,^22^ Pakistan^18^ and Nigeria,^33^ personal interests have been found to be the most important factor influencing the choice of speciality. We report a similar trend in our study, although benefits to patient ranked second to personal interest. Interestingly, and contrary to other views,^34^ factors such as financial rewards, social status and lifestyle are not viewed as being premium reasons for the choice of speciality. This again may translate to the fact that wage disparities amongst public sector health workers are minimal, irrespective of the chosen speciality.

In order to enhance the spread of speciality manpower development to meet the goal of healthcare for all, as well as to target visible manpower deficiencies in certain specialities and careers in basic medical sciences and/or the research faculty, there is the need for a more focused national medical education policy. This should address the need or mechanisms for determining the number and areas for specialities in medical education, especially for government-funded public institutions.

## Strengths and limitations

The strengths of our study include an acceptable response rate with over 80% of the target group responding. We chose first-year students to learn about their initial thoughts on career choice and our plan is to repeat the study with the same students as they progress yearly, to identify when changes occur. Our study extends knowledge about factors influencing choice of speciality by paying particular attention to factors that are amenable to change and to variation in these factors across disciplines. The study's weaknesses include that the reasons for non-response are not known.

## Conclusion

This study has shown that a high proportion of the first-year medical students in UKZN are inclined to specialise in clinical disciplines, with the majority preferring the public sector as first choice. There is also a positive indication that the majority of students intend to remain within the country for either their medical practice or to pursue their careers in their intended specialisation, which is very encouraging and needs to be supported and amplified if the goals of adequate healthcare delivery are to be met in South Africa.

## References

[1] Newton DA, Grayson MSand Whitley TW What predicts medical student career choice? J Gen Intern Med. 1998;13(3):200–203. http://dx.doi.org/10.1046/j.1525-1497.1998.00057.x, PMCid:1496924 9541378 10.1046/j.1525-1497.1998.00057.x PMC1496924

[2] Aslam M, Ali A, Taj T, Badar N, Mirza W, Ammar A, et al Specialty choices of medical students and house officers in Karachi, Pakistan. East Mediterr Health J. 2011;17(1):74–79. PMid:21735806 21735806

[3] Campos-Outcalt D, Senf J, Watkins AJ, Bastacky S The effects of medical school curricula, faculty role models, and biomedical research support on choice of generalist physician careers: a review and quality assessment of the literature. Acad Med. 1995;70(7):611–619. http://dx.doi.org/10.1097/00001888-199507000-00012, PMid:7612127 7612127 10.1097/00001888-199507000-00012

[4] Matorin AA, Venegas-Samuels K, Ruiz P, Butler PM, Abdulla A U.S. medical students’ choice of careers and its future impact on health care manpower. J Health Hum Serv Adm. 2000;22(4):495–509. PMid:11211559 11211559

[5] Newton DA, Grayson MS Trends in career choice by US medical school graduates. JAMA. 2003;290(9): 1179–1182. http://dx.doi.org/10.1001/jama.290.9.1179, PMid:12953000 12953000 10.1001/jama.290.9.1179

[6] Fisher ES, Wennberg DE, Stukel TA, Gottlieb DJ, Lucas FL, Pinder EL The implications of regional variations in Medicare spending. Part 2: health outcomes and satisfaction with care. Ann Intern Med. 2003;138(4):288–298. http://dx.doi.org/10.7326/0003-4819-138-4-200302180-00007, PMid:12585826 12585826 10.7326/0003-4819-138-4-200302180-00007

[7] Barshes NR, Vavra AK, Miller A, Brunicardi FC, Goss JA, Sweeney JF General surgery as a career: a contemporary review of factors central to medical student specialty choice. J Am Coll Surg. 199(5):792–799. http://dx.doi.org/10.1016/j.jamcollsurg.2004.05.281, PMid:15501122 15501122 10.1016/j.jamcollsurg.2004.05.281

[8] Cochran A, Melby S, Neumayer LA An internet-based survey of factors influencing medical student selection of a general surgery career. Am J Surg. 2005;189(6):742–746. http://dx.doi.org/10.1016/j.amjsurg.2005.03.019, PMid:15910730 15910730 10.1016/j.amjsurg.2005.03.019

[9] Ruhnke GW Physician supply and the shifting paradigm of medical student choice. JAMA. 1997;277(1):70–71. http://dx.doi.org/10.1001/jama.277.1.70, PMid:8980216 8980216 10.1001/jama.277.1.70

[10] Sierles FS, Dinwiddie SH, Patroi D, Atre-Vaidya N, Schrift MJ, Woodard JL Factors affecting medical student career choice of psychiatry from 1999 to 2001. Acad Psychiatry. 2003;27(4):260–268. http://dx.doi.org/10.1176/appi.ap.27.4.260 14754849 10.1176/appi.ap.27.4.260

[11] Reed VA, Jernstedt GC, Reber ES Understanding and improving medical student specialty choice: a synthesis of the literature using decision theory as a referent. Teach Learn Med. 2001 Spring;13(2):117–129. http://dx.doi.org/10.1207/S15328015TLM1302_7, PMid:11302032 11302032 10.1207/S15328015TLM1302_7

[12] De Vries E, Irlam J, Couper I, Kornik S Career plans of final-year medical students in South Africa. S Afr Med J. 2010;100(4):227–228. PMid:20459968 20459968 10.7196/samj.3856

[13] Harris MG, Gavel PH, Young JR Factors influencing the choice of specialty of Australian medical graduates. Med J Aust. 2005;183(6):295–300. PMid:16167868 16167868 10.5694/j.1326-5377.2005.tb07058.x

[14] Nieman LZ, Holbert D, Bremer CC, Nieman LJ Specialty career decision making of third-year medical students. Fam Med. 1989;21(5):359–363. PMid:2792607 2792607

[15] Friedman CTH, McGuire FL A survey of a freshman medical student class: will psychiatry recruit well into the 1980s? J Psychiatr Education. 1982;5:115–122.

[16] Lambert TW, Goldacre MJ, Edwards C, Parkhouse J Career preferences of doctors who qualified in the United Kingdom in 1993 compared with those of doctors qualifying in 1974, 1977, 1980 and 1983. BMJ. 1996;313(7048):19–24. http://dx.doi.org/10.1136/bmj.313.7048.19, PMid:8664763, PMCid:2351449 8664763 10.1136/bmj.313.7048.19 PMC2351449

[17] Halpern N, Bentov-Gofrit D Matot Iand Abramowitz MZ(2011). The effect of integration of non-cognitive parameters on medical students’ characteristics and their intended career choices. Isr Med Assoc J. 2011;13(8):488–493. PMid:21910374 21910374

[18] Huda N, Yousuf S Career preference of final year medical students of Ziauddin Medical University. Educ Health (Abingdon). 2006;19(3):345–353. http://dx.doi.org/10.1080/13576280600984087, PMid:17178516 17178516 10.1080/13576280600984087

[19] Akinyinka OO, Ohaeri JU, Asuzu MC Beliefs and attitudes of clinical year students concerning medical specialties: an Ibadan medical school study. Afr J Med Med Sci. 1992;21(2):89–99. PMid:1308088 1308088

[20] Ihekweazu C, Anya I, Anisoke E Nigerian medical school graduates: where are they now? Lancet. 2005;365(9474):1847–1848. http://dx.doi.org/10.1016/S0140-6736(05)66612-3 15924978 10.1016/S0140-6736(05)66612-3

[21] Burch VC, McKinley D, van Wyk J, Kiguli-Walube S, Cameron D, Cilliers FJ, et al Career intentions of medical students trained in six sub-Saharan African countries. Educ Health (Abingdon). 2011;24(3):614. 22267357

[22] Dambisya Y Career intentions of UNITRA medical students and their perceptions about the future. Educ Health (Abingdon). 2003;16(3):286–297. http://dx.doi.org/10.1080/13576280310001607442, PMid:14741877 14741877 10.1080/13576280310001607442

[23] Yeganeh-Arani E, Chandratilake M, Muula AS Factors affecting career preferences of medical students at the College of Medicine, Malawi. S Afr Med J. 2012;102(4):249–251. PMid:22464509 22464509

[24] Association of American Medical Colleges Americans still advise their kids to ‘Be a Doctor’. New Gallup May 19, 2005. [page on Internet]. [c2005] [cited 2012 Dec 12]. Available from https://www.aamc.org/newsroom

[25] Ebomoyi MI, Agoreyo FD Preclinical students’ perceptions of their courses and preclinical specialty choice. IJMBR. 2007;6(1&2):47–58.

[26] Osipow SH Assessing career indecision. J Vocat Behav. 1999;55(1):147–154. http://dx.doi.org/10.1006/jvbe.1999.1704

[27] Kelly KR, Lee W-C Mapping the domain of career decision problems. J Vocat Behav. 61(2):302–326. http://dx.doi.org/10.1006/jvbe.2001.1858

[28] World Health Organization The World Health Report 2006 – working together for health. [page on Internet]. [c2006] [cited 2008 Aug 10]. Available from http://www.who.int/whr/2006/en

[29] Borges NJ Behavioral exploration of career and specialty choice in medical students. Career Dev Q. 2007;55(4):351–358. http://dx.doi.org/10.1002/j.2161-0045.2007.tb00089.x

[30] Mullan F, Frehywot S, Omaswa F, Buch E, Chen C, Greysen SR, et al Medical schools in sub-Saharan Africa. Lancet. 2011;377(9771):1113–1121. http://dx.doi.org/10.1016/S0140-6736(10)61961-7 21074256 10.1016/S0140-6736(10)61961-7

[31] Oyebola DD, Adewole OE Preference of preclinical medical students for medical specialties and basic medical sciences. Afr J Med Med Sci. 1998;27(3–4):209–212. PMid:10497651 10497651

[32] Anand MK, Raibagkar CJ, Ghediya SV, Singh P Anatomy as a subject and career option in view of medical students in India. J Anat Soc India. 2004;53(1):10–14.

[33] Odusanya OO, Nwawolo CC Career aspirations of house officers in Lagos, Nigeria. Med Educ. 2001;35(5):482–487. http://dx.doi.org/10.1046/j.1365-2923.2001.00896.x 11328519 10.1046/j.1365-2923.2001.00896.x

[34] Aydin S, Yaris F, Sahin ME, Ozer C, Ozkomur E Students’ perception of their undergraduate medical education. Saudi Med J. 2005;26(9):1484–1486. PMid:16155682 16155682

